# On‐Line Analysis of Cigarette Smoke Based on Microwave Plasma Torch Mass Spectrometry

**DOI:** 10.1002/open.202400013

**Published:** 2024-06-14

**Authors:** He Tang, Kailong Yuan, Fengjian Chu, Xiaobing Zhang, Qi Li, Qi Chen, Hongru Feng, Yuanjiang Pan

**Affiliations:** ^1^ China Tobacco Zhejiang Industrial Co. Ltd. Hangzhou Zhejiang 310008 China; ^2^ Department of Chemistry Zhejiang University Hangzhou Zhejiang 310027 China

**Keywords:** Cigarette smoke, Microwave plasma torch, polycyclic aromatic hydrocarbons

## Abstract

Cigarette smoke contains a large number of chemicals, including both flavor components and harmful substances. The mainstream smoke (MSS) generated by smoking is directly inhaled by individuals, making it crucial to establish an effective method for smoke detection and analysis. One promising technique for analyzing smoke is MPT‐MS (Microwave plasma torch mass spectrometry). This approach offers several advantages in accurately detecting the composition of cigarette smoke. By combining MPT‐MS with a smoke pumping device, we can achieve real‐time online detection of smoke components. We successfully detected 22 flavor compounds present in the smoke. These compounds contribute to the distinct taste of cigarettes. Moreover, we identified 2 polycyclic aromatic hydrocarbons (PAHs) in the smoke. PAHs are known carcinogens and are of great concern in terms of their potential health risks. The successful detection and identification of flavor compounds and PAHs using our method confirm the online detection capability of MPT‐MS. This approach provides an efficient and reliable means for analyzing the complex composition of cigarette smoke. By utilizing MPT‐MS, we can gain valuable insights into the chemical composition of cigarette smoke and can inform the development of strategies and policies aimed at reducing the harmful effects of smoking and protecting public health.

## Graphical abstract

Cigarette smoke contains a large number of flavor components and harmful substances, so it is crucial to establish an effective method for smoke detection and analysis. This article provides a method for on‐line analysis of smoke with MPT‐MS (Microwave plasma torch mass spectrometry), and achieved detection of 22 flavor compounds and 2 polycyclic aromatic hydrocarbons (PAHs) in the smoke.

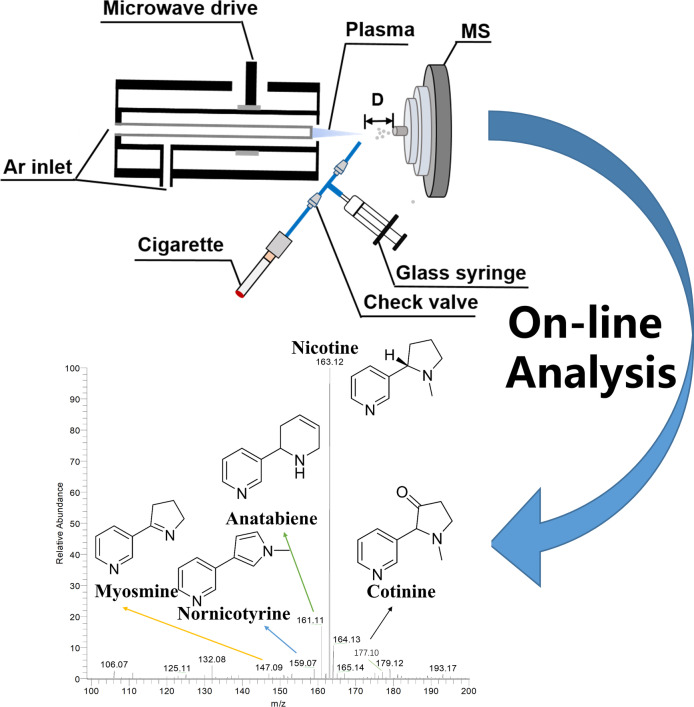

## 1 Introduction

The cigarette industry is a globally significant industry that continues to evolve. It has established thorough procedures encompassing tobacco cultivation, processing, and cigarette preparation. However, due to the intricate composition of tobacco, there are thousands of compounds present in the smoke produced by the combustion of cigarettes, which are subsequently inhaled into the human body. The cigarettes smoke enters the body via the filter is commonly known as mainstream smoke. The composition of mainstream smoke significantly affects the taste of cigarettes, with aldehydes and ketones playing a crucial role in the flavor of cigarettes,^[1]^ and some components have significant impact on human health, such as polycyclic aromatic hydrocarbons (PAHs) and nitrosamine compounds, which are typical carcinogens.[^1^, ^2^] Analyzing the composition of cigarette smoke can effectively control cigarette quality, while testing harmful substances can monitor tobacco safety. Consequently, there is still a need for continuous improvement in the monitoring of quality and safety throughout the cigarette production process, which is also beneficial for the development of the tobacco industry and human health.

The detection of cigarette smoke components often requires pre‐treatment such as gas trapping,^[3]^ solvent extraction, or solid‐phase microextraction.^[4]^ The separated components are then analyzed using chromatography and mass spectrometry, such as gas chromatography‐mass spectrometry (GC‐MS) and liquid chromatography‐mass spectrometry (LC–MS).[^4^, ^5^] These methods have good reproducibility and accuracy, but they need long analysis period and cannot achieve online rapid detection of samples. It is of great significance to establish online detection methods for cigarette smoke. Currently, online detection device for cigarette smoke have been reported, such as vacuum ultraviolet photoionization time‐of‐flight mass spectrometry (VUV‐PI‐TOFMS).^[6]^ However, the device is complicated, and the mass spectrometry signal response of crucial constituents in cigarette smoke lacks clarity and distinction.

The emergence of Desorption Electrospray Ionization (DESI)^[7]^ and Direct Analysis in Real Time (DART)^[8]^ signifies the prosperity of Ambient Ionization Mass Spectrometry (AIMS). AIMS enables rapid analysis of substances without or with little sample pre‐treatment, and achieves direct environmental sample analysis.^[9]^ This provides ways for the development of high‐throughput and high‐sensitivity mass spectrometry analysis methods.^[10]^

Recently, AIMS has been used for rapid detection of cigarette smoke components, including liquid sampling‐atmospheric pressure glow discharge mass spectrometry (LS‐APGD‐MS),^[11]^ desorption corona beam ionization (DCBI‐MS)^[12]^ and DART‐MS.[^13^, ^14^] Microwave plasma torch mass spectrometry (MPT‐MS) is AIMS that has developed in the past decade. MPT generator is small in size, low in power, and easy to operate. It can generate a strong excitation plasma beam, enabling gas, liquid, and solid samples to be ionized in the atmospheric environment without any pre‐treatment, and then analyzed by mass spectrometry.^[15]^ MPT can produce water radical cations(H_2_O^+^⋅), hydroxyl radicals (HO^•^) and reactive oxygen species,[^16^, ^17^] resulting in sample ionization to produce signal ions such as [M+H]^+^, [M+H+O]^+^, [M+H+2O]^+^. MPT‐MS allows us to predict the oxidation product of some pollutant and can be used for pollutant identification,^[17]^ and it can also help us find some characteristic oxidation products in non‐target research. Currently, MPT‐MS has achieved low detection limits and shows good performance in the detection of trace compounds in the environment or food,[^16^, ^18^, ^19^, ^20^, ^21^] detection of natural products[^22^, ^23^] and drug.^[24]^

In this research, we developed a smoke sampling device to replicate the inhalation and exhalation process of cigarette smoke by human beings, enabling online real‐time detection of smoke through microwave plasma torch mass spectrometry. A total of 22 flavor compounds were detected, the results from MPT‐MS testing were in line with the results obtained from the analysis of smoke extracts using electrospray ionization mass spectrometry (ESI‐MS). And 2 PAHs were found in cigarette smoke by MPT‐MS analysis. This demonstrates the online analyzing ability of our device combined with MPT‐MS.

## 2 Results and Discussion

### 2.1 Optimization of MPT‐MS Condition

**Figure 1:**
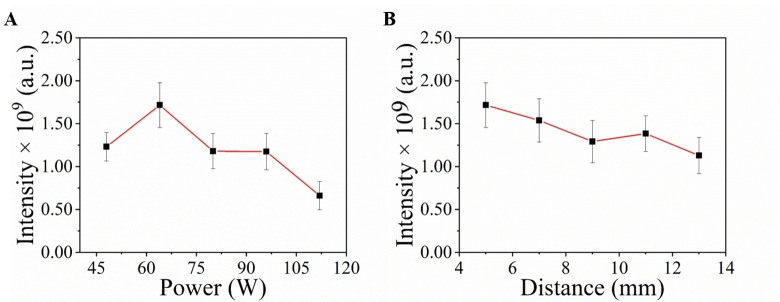
Optimization of (A) MPT power, (B) distance between MPT and MS.

The power of MPT and distance between MPT and MS are important parameters. Nicotine is the primary component of cigarette smoke, and exhibits the highest intensity in MS. Therefore, we select nicotine intensity with the change of power and distance as the reference for optimizing the analysis conditions(Figure 1). Initially, we set the distance between MPT and MS to 5 mm and adjusted the power of MPT to evaluate its impact. With energy increases, the compounds may be fragmented, thus reducing the MS intensity. It was observed that the highest intensity of nicotine was achieved at a power of 64 W, thus establishing it as the optimal condition. Subsequently, we proceeded to optimize the distance between MPT and MS. Although the MS intensity showed minimal variation with changing distance, there was a gradual decline trend as the distance increased. This phenomenon may be attributed to the rapid dispersion of smoke in the air, while the flow of MPT effectively carries ionized smoke into MS. These observations highlight the stability of MPT in testing smoke samples and its strong resistance to interferences.

### 2.2 On‐Line Analysis of Cigarette Smoke

To validate the partial components in the smoke, we employed a gas washing bottle to collect the smoke components., a polar solvent ethanol, and a non‐polar solvent DCM, were selected to capture the smoke. However, the efficiency of solvent extraction for smoke is low, so each suction had to be performed slowly, allowing the solution to fully extract the smoke before the next suction, resulting in a slow process. Once the collection was completed, we conducted ESI‐MS analysis. In the low molecular weight region (m/z<100), numerous isomers of smoke components were present, requiring further structural identification. Compound with a mass range greater than 100 were identified based on the [M+H]^+^ ion. Through ESI, we were able to identify more than twenty compounds (Table S1 and S2). While different solvents were utilized for collection, the results remained consistent, with only slight variations in the mass spectrometry responses of different compounds. DCM in MPT resulted in complicate spectrum, so we employed ethanol extraction to be analyzed in MPT‐MS, which obtained consistent results with ESI‐MS (Table S3).

**Figure 2:**
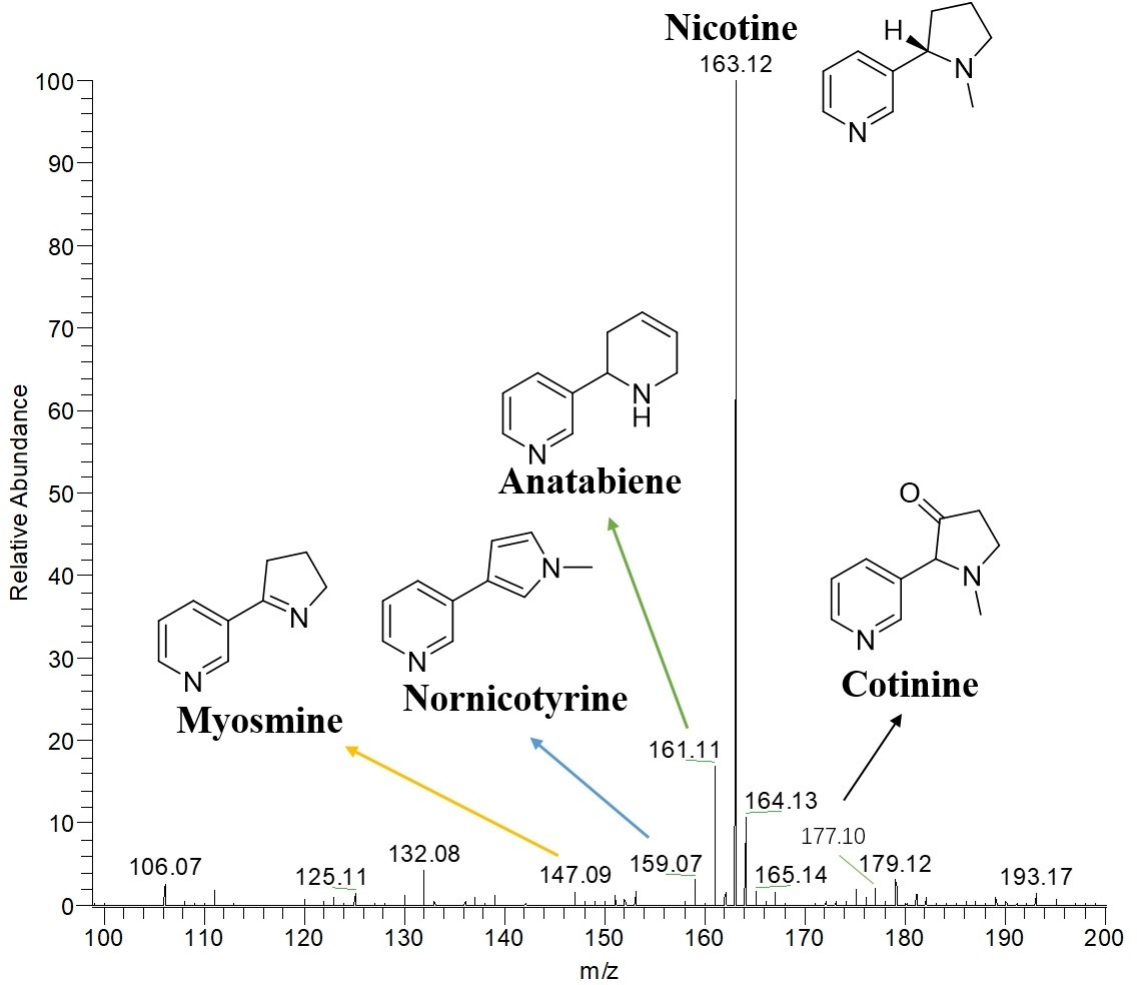
Mass spectrum of cigarette components detected online.

We proceeded to perform MPT‐MS analysis of smoke. The utilization of MPT for online smoke analysis allows for the analysis of all the compounds identified in ESI. As anticipated, nicotine and its analogues (Figure 2 and Table 1)), namely myosmine, nornicotine, anatabine, and cotinine, exhibited the highest response. Additionally, other alkaloids, aldehyde, and ketone aroma components, such as benzaldehyde, pyridine‐3‐aldehyde, 2,6‐lutidine, phenethyl alcohol, 3‐ethyl‐4‐methyl‐3‐pyrrolin‐2‐one, 1‐ethyl‐3‐hydroxypiperidine, 2‐Methylindole, cinnamaldehyde, 3‐ethyl‐4‐methyl‐pyrrole‐2,5‐dione, vanillylacetone, 2‐phenyl‐2‐butenal, 5‐hydroxy‐2‐methylindole, vanillin, nornicotyrine, 5,6‐dimethyl‐1H‐benzo[d]imidazol‐2(3H)‐one, 3,4‐dimethoxybenzaldehyde, 5‐(1‐piperidyl)furan‐2‐carbaldehyde, and syringyl ethene, could also be detected. The MS^2^ spectra of 14 compounds were given(Figure S4–17) and compared our compounds with secondary mass spectra from multiple databases (HMDB, PlantCyc, NPA, NANPDB, COCONUT, UNPD, etc.) using MS‐Finder. In the range of m/z<100, there are also a large number of compounds that can be detected. However, compounds in this range have multiple isomers, which need to be differentiated through the comparison of multi‐stage mass spectra with standard substances. This may be our subsequent work. Compounds in the range of 100<m/z<200 showed highest intensity so our analysis method are most suitable for analyzing this range. Compounds with m/z>200 have weaker signals or may not be detected at all. This may be because cigarettes themselves contain a high content of small‐molecule compounds, which can suppress the ionization of low‐content compounds. It is also possible that the plasma has caused the fragmentation of compounds with relatively large molecular weights, and only their characteristic fragment ions can be seen in the mass spectra, such as α/β‐cembranoids^[23]^ (Figure S2, S3).

**Table 1:** Cigarette smoke flavor components.

Compound number	Compound name	Relative molecular weight	Molecular Formula	Calculated [M+H]^+^	Measured [M+H]^+^	Mass error δ (×10^−6^)
1	Benzaldehyde	106.0419	C_7_H_6_O	107.0497	107.0495	−1.87
2	Pyridine‐3‐aldehyde	107.0371	C_6_H_5_NO	108.0449	108.0447	−1.85
3	2,6‐Lutidine	107.0735	C_7_H_9_N	108.0813	108.0810	−2.78
4	Phenethyl alcohol	122.0732	C_8_H_10_O	123.0810	123.0807	−2.44
5	3‐Ethyl‐4‐methyl‐3‐pyrrolin‐2‐one	125.0841	C_7_H_11_NO	126.0919	126.0917	−1.59
6	1‐Ethyl‐3‐hydroxypiperidine	129.1154	C_7_H_15_NO	130.1232	130.123	−1.54
7	2‐Methylindole	131.0735	C_9_H_9_N	132.0813	132.0811	−1.51
8	Cinnamaldehyde	132.0575	C_9_H_8_O	133.0653	133.0654	0.75
9	3‐ethyl‐4‐methyl‐pyrrole‐2,5‐dione	139.0633	C_7_H_9_NO_2_	140.0712	140.0709	−2.14
10	2‐Phenyl‐2‐butenal	146.0732	C_10_H_10_O	147.0810	147.0811	0.68
11	Myosmine	146.0844	C_9_H_10_N_2_	147.0922	147.092	−1.36
12	5‐Hydroxy‐2‐methylindole	147.0684	C_9_H_9_NO	148.0762	148.076	−1.35
13	Nornicotine	148.1000	C_9_H_12_N_2_	149.1079	149.1077	−1.34
14	Vanillin	152.0473	C_8_H_8_O_3_	153.0552	153.0550	−1.31
15	Nicotyrine	158.0844	C_10_H_10_N_2_	159.0922	159.0919	−1.89
16	Anatabine	160.2200	C_10_H_12_N_2_	161.1079	161.1077	−1.24
17	5, 6‐Dimethyl‐1H‐benzo[d]imidazol‐2(3H)‐one	162.0793	C_9_H_10_N_2_O	163.0871	163.0872	0.61
18	Nicotine	162.2360	C_10_H_14_N_2_	163.1235	163.1232	−1.84
19	3,4‐Dimethoxybenzaldehyde	166.0630	C_9_H_10_O_3_	167.0708	167.0706	−1.20
20	Cotinine	176.0950	C_10_H_12_N_2_O	177.1028	177.1025	−1.69
21	5‐(1‐piperidyl)furan‐2‐carbaldehyde	179.0946	C_10_H_13_NO_2_	180.1025	180.1022	−1.67
22	Syringlyethene	180.0786	C_10_H_12_O_3_	181.0865	181.0863	−1.10

PAHs have low concentrations in cigarette smoke^[25]^ and can be suppressed by high intensity ions, such as alkaloids, inhibiting signals. Based on our method, we have identified two PAHs in the cigarette smoke, acenaphthylene and acenaphthene (Table 2). Other PAHs maybe enveloped by peaks of other compounds and cannot be distinguished without sufficient resolution. This demonstrated the capability of MPT‐MS in detecting PAHs in cigarette smoke and with an increase in mass spectrometer resolution, maybe we can detect more PAHs. The MS intensity of compounds detected were all listed in Table 3.

**Table 2:** PAHs in cigarette smoke.

Compound number	Compound name	Relative molecular weight	Molecular Formula	Measured [M+H]^+^	Mass error δ (×10^−6^)
1	Acenaphthylene	152.0626	C_12_H_8_	152.0624	−1.32
2	Acenaphthene	154.0783	C_12_H_10_	154.0780	−1.95

**Table 3:** Cigarette smoke components intensity.

Compound number	Compound name	Relative molecular weight	Molecular Formula	Average Intensity (a. u.)	Relative Intensity (%)
1	Benzaldehyde	106.0419	C_7_H_6_O	4.5E+06	0.25 %
2	Pyridine‐3‐aldehyde	107.0371	C_6_H_5_NO	2.1E+07	1.13 %
3	2,6‐Lutidine	107.0735	C_7_H_9_N	5.1E+07	2.83 %
4	Phenethyl alcohol	122.0732	C_8_H_10_O	1.4E+07	0.76 %
5	3‐Ethyl‐4‐methyl‐3‐pyrrolin‐2‐one	125.0841	C_7_H_11_NO	1.1E+07	0.63 %
6	1‐Ethyl‐3‐hydroxypiperidine	129.1154	C_7_H_15_NO	3.3E+06	0.18 %
7	2‐Methylindole	131.0735	C_9_H_9_N	7.9E+07	4.35 %
8	Cinnamaldehyde	132.0575	C_9_H_8_O	2.7E+06	0.15 %
9	3‐ethyl‐4‐methyl‐pyrrole‐2,5‐dione	139.0633	C_7_H_9_NO_2_	4.2E+06	0.23 %
10	2‐Phenyl‐2‐butenal	146.0732	C_10_H_10_O	3.3E+06	0.18 %
11	Myosmine	146.0844	C_9_H_10_N_2_	9.2E+07	5.04 %
12	5‐Hydroxy‐2‐methylindole	147.0684	C_9_H_9_NO	3.4E+07	1.87 %
13	Nornicotine	148.1000	C_9_H_12_N_2_	4.6E+07	2.54 %
14	Vanillin	152.0473	C_8_H_8_O_3_	5.9E+06	0.32 %
15	Nicotyrine	158.0844	C_10_H_10_N_2_	4.3E+07	2.35 %
16	Anatabine	160.2200	C_10_H_12_N_2_	2.5E+08	13.69 %
17	5, 6‐Dimethyl‐1H‐benzo[d]imidazol‐2(3H)‐one	162.0793	C_9_H_10_N_2_O	3.5E+07	1.93 %
18	Nicotine	162.2360	C_10_H_14_N_2_	1.8E+09	100.00 %
19	3,4‐Dimethoxybenzaldehyde	166.0630	C_9_H_10_O_3_	5.7E+06	0.32 %
20	Cotinine	176.0950	C_10_H_12_N_2_O	7.1E+07	3.89 %
21	5‐(1‐piperidyl)furan‐2‐carbaldehyde	179.0946	C_10_H_13_NO_2_	2.4E+07	1.31 %
22	Syringlyethene	180.0786	C_10_H_12_O_3_	6.5E+06	0.36 %
23	Acenaphthylene	152.0626	C_12_H_8_	2.4E+05	0.01 %
24	Acenaphthene	154.0783	C_12_H_10_	3.5E+05	0.02 %

**Figure 3:**
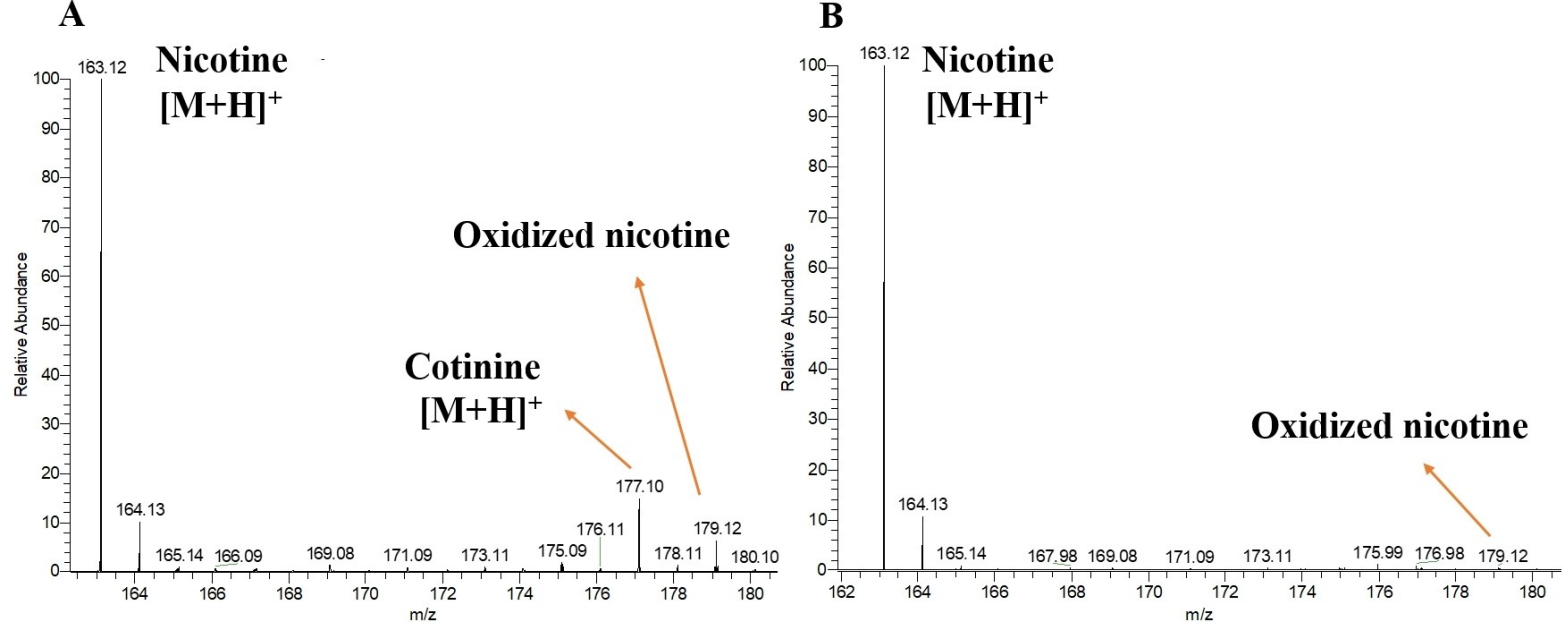
Mass spectrum of (A) cigarette smoke analyzed by MPT‐MS (B) ethanol extraction of cigarette smoke analyzed by ESI‐MS.

Through MS^1^ (Figure 3) and MS^2^ analysis (Figure S1), it is inferred that both ESI‐MS and MPT‐MS can detect cotinine and the oxidation products of nicotine, but the intensity is obviously higher in MPT‐MS. As mentioned above, MPT can produce hydroxyl radical (HO⋅) and a large number of reactive oxygen species. Besides the oxidation of nicotine during cigarette combustion, a small amount of oxidation obviously occurs in MPT, leading to the higher oxidation products.

**Figure 4:**
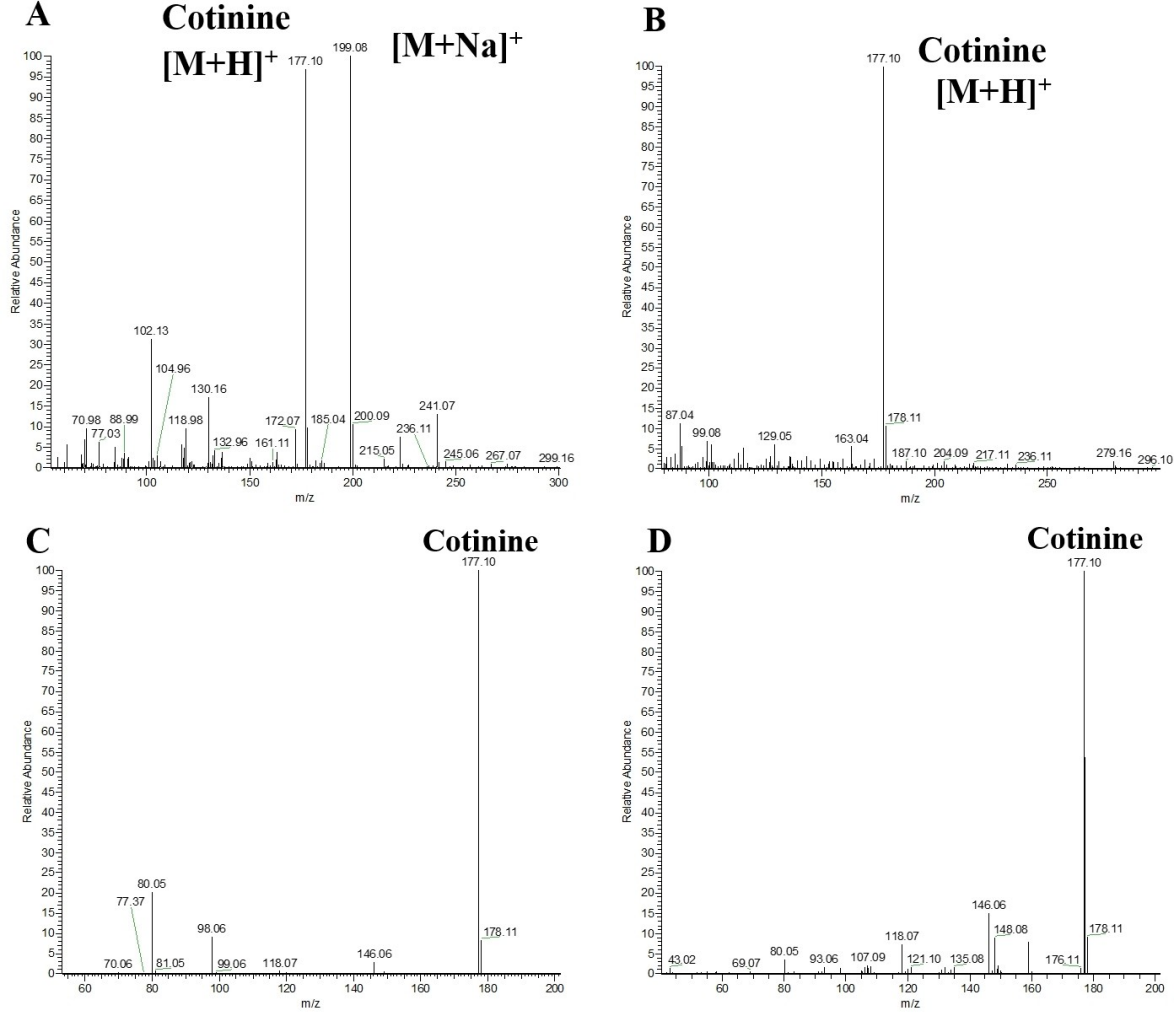
Spectrum of (A) MS^1^ of cotinine standard in ESI‐MS. (B) MS^1^ of cotinine in MPT‐MS. (C) MS^2^ of cotinine standard in ESI‐MS. (D) MS^2^ of cotinine in MPT‐MS.

By comparing the MS^2^ of the standard solution, we have confirmed that m/z 177.10 in cigarette smoke is cotinine (Figure 4). In the MPT‐MS spectra, cotinine shows a significantly higher response. And in the ESI‐MS, the cotinine solution exhibits a [M+Na]^+^ peak, while no [M+Na]^+^ peak is observed in the MPT‐MS. This simplifies the spectra and facilitates the detection of the compound in complex mixtures. In conclusion, MPT‐MS demonstrates high ionization efficiency and helps us identify oxidation products, offering greater advantages for the detection of smoke components compared to other methods, and enabling online monitoring.

## 3 Materials and Methods

### 3.1 Instruments

**Figure 5:**
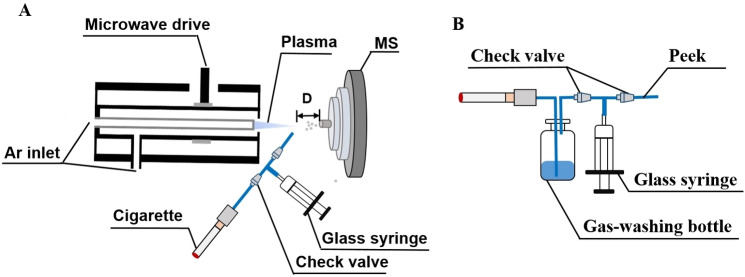
Schematic diagram of (A) online analysis of smoke composition by MPT‐MS and (B) cigarette smoke extraction device.

The microwave plasma torch (MPT) ion source, as shown in Figure 5, was built in our laboratory. The MPT generator used argon as the working gas and plasma was excited from 0 to 200 W within the frequency range of 2450 MHz using a microwave generator. Argon was controlled by a rotor flowmeter, its flow rate could be adjusted at 2 mL/min. The injection was placed at an angle of 45° to the plasma torch, and the front end was 5 mm away from the torch. The entire device was supported by a multi‐axis platform, which was used to control the distance D between the MPT front end and the mass spectrometer inlet.

Mass spectrometer: Orbitrap Exploris 120 (Thermo Fisher Scientific, San Jose, CA), which is used for real‐time compound detection. Connecting MS with ultra performance liquid chromatography Vanquish™ system (Thermo Fisher Scientific, CA, USA) to do ESI analysis with conditions: flow rate 0.2 mL/min with mobile phase ratio MeOH: H_2_O 1 : 1; ion spray voltage: 3500 V; ion transfer tube temperature: 320 °C;Vaporizer temperature 275 °C; RF lens: 70 %; sheath gas 35 Arb; aux gas 7 Arb; injection time 100 ms. Tandem MS conditions: Nitrogen was introduced as the collision gas and collision energy was 40 %. All experiments were conducted in positive ion mode with a resolution of 30000 and a mass range of m/z 50–750. Data acquisition was carried out using the Xcalibur 2.2 SP1 software (Thermo Fisher Scientific, San Jose, CA).

The smoke suction device consists of a Peek tube, two check valves, and a 100 mL glass syringe. The smoke collection device, based on the suction device, adds a gas washing bottle before the first check valve. A pump (ISPLab01, DK Infusetek Technology, Shanghai) is used to pump in and drain smoke in a set rate.

### 3.2 Materials

Cigarettes used for experiments came from a common brand (China). Cotinine (98 %) were purchased from Meryer Biochemical Technology (Shanghai, China). HPLC‐grade ethanol and methanol, were purchased from Sigma–Aldrich (St. Louis, MO, USA). Water used for the HPLC mobile phase was purchased from A.S. Watson Group Ltd. (Hong Kong, China). Dichloromethane (Analytical Reagent) was purchased from Sinopharm Chemical Reagent Co., Ltd (Shanghai, China). Argon (99.99 % purity), were purchased from Zingergas Co., Ltd (Hangzhou, China). Nitrogen were generated by NiGen LCMS 40–1from Claind Co., Ltd (Villa Carlotta, Italy)

### 3.3 Smoke Online Analysis and Capture Method

Online analysis method: 10 mL of smoke is drawn each time and then pushed out at a rate of 2 mL/s towards the front end of the plasma torch using a pump.

Smoke capture method: as shown in Figure 5, a device was used for capturing the smoke. A gas wa1shing bottle was added between the syringe and the cigarette. To capture the smoke of an entire cigarette, 50 mL of solvent was added, and the syringe was used to draw until the smoke is captured into the 50 mL of solvent. The solvent is polar organic solvent ethanol (EtOH) and a non‐polar organic solvent dichloromethane (DCM).

### 3.4 Cotinine Standard Preparation

Cotinine was dissolved in methanol with concentration of 10 ppm to get 1 mL solution.

## 4 Conclusions

So far, many analytical detection techniques have been applied in the tobacco industry. Among them, GC‐MS is the most commonly used technique for detecting tobacco components, based on the abundance of small molecular compounds in tobacco. However, whether it is GC‐MS or other techniques, it usually requires a trapping process or other complex procedures. In contrast, AIMS is convenient and fast for tobacco smoke detection, and it can reflect the real‐time composition of tobacco or smoke compounds, achieving in situ or real‐time analysis. Although several techniques have been used for smoke detection, our proposed online detection method based on MPT‐MS is easy to operate, with high‐quality spectra, and suitable for high‐throughput smoke component detection. It has certain advantages compared to other techniques.

In this research, we developed an on‐line detection method for analyzing mainstream cigarette smoke by combining a smoke pumping device with MPT‐MS. The cigarette smoke is directly introduced to the front end of the MPT, where it is ionized and enters the mass spectrometry for analysis. This eliminates the need for sample preparation. Comparing our method with ESI‐MS, MPT‐MS analysis covers the compounds that can be detected using ESI‐MS. Through these ways we detected 22 flavor compounds and 2 PAHs, which are helpful in evaluating quality and safety of cigarettes. Additionally, MPT has a wide range of ionization ability from non‐polar compound to polar compound and provides more information through mass spectrometry for they can ionize PAHs. Therefore, we plan to conduct further research on the ionization and fragmentation mechanism of MPT in order to detect more compounds in cigarette smoke and make some contribution to cigarette quality control. In conclusion, this study presents a rapid and efficient on‐line analysis method for studying the chemical composition of cigarette smoke.

Further research and advancements in smoke detection and analysis techniques are essential for continuously improving our understanding of tobacco smoke composition. This knowledge will aid in the development of more effective measures to control and regulate the tobacco industry, ultimately promoting public health and well‐being.

## Conflict of Interests

The authors declare no conflict of interest.

5

## Supporting information

As a service to our authors and readers, this journal provides supporting information supplied by the authors. Such materials are peer reviewed and may be re‐organized for online delivery, but are not copy‐edited or typeset. Technical support issues arising from supporting information (other than missing files) should be addressed to the authors.

Supporting Information

## Data Availability

The data that support the findings of this study are available from the corresponding author upon reasonable request.
