# Supplementary material for: On‐Line Analysis of Cigarette Smoke Based on Microwave Plasma Torch Mass Spectrometry
Source: ChemistryOpen. 2024 Jun 14;13(9):e202400013. doi: 10.1002/open.202400013 (PMC11467726; doi:10.1002/open.202400013)
Supplement: Supplementary file 1 — Supporting Information [file OPEN-13-e202400013-s001.pdf]

ChemistryOpen

Supporting Information

On-Line Analysis of Cigarette Smoke Based on Microwave Plasma Torch Mass Spectrometry

He Tang,* Kailong Yuan,* Fengjian Chu,* Xiaobing Zhang,* Qi Li,* Qi Chen,* Hongru Feng,* and Yuanjiang Pan*

On-line analysis of cigarette smoke based on microwave plasma torch mass spectrometry

He Tang[1,2,†], Kailong Yuan[1,†], Fengjian Chu[2], Xiaobing Zhang[1], Qi Li[1], Qi Chen[2], Hongru Feng*, Yuanjiang Pan*

1 Zhejiang China Tobacco Industrial Co. , Ltd.， Hangzhou, Zhejiang, 310008, China; henryt98@163.com (H.T.); yuankailong@zjtobacoo.com (K.Y.); zhangxb@zjtobacoo.com (X.Z.); liqi@zjtobacoo.com (Q.L.)

2 Department of Chemistry, Zhejiang University, Hangzhou, Zhejiang, 310027, China; (F.C.); 3200102241@zju.edu.cn (Q.C.)

* Correspondence: fenghongru@zju.edu.cn (H.F.); panyuanjiang@zju.edu.cn (Y.P.)

† These authors contributed equally to this work

Table S1. Smoke extracts components（EtOH, ESI）

Compound number	Compound name	Relative molecular weight	Molecular Formula	Calculated $[M+H]^+$	Measured $[M+H]^+$	Mass error δ ($\times 10^{-6}$)
1	Benzaldehyde	106.0419	C_7H_6O	107.0497	107.0493	-3.74
2	Pyridine-3-aldehyde	107.0371	C_6H_5NO	108.0449	108.0446	-2.78
3	2,6-Lutidine	107.0735	C_7H_9N	108.0813	108.081	-2.78
4	Phenethyl alcohol	122.0732	$C_8H_{10}O$	123.0810	123.0807	-2.44
5	3-Ethyl-4-methyl-3-pyrrolin-2-one	125.0841	$C_7H_{11}NO$	126.0919	126.0917	-1.59
6	1-Ethyl-3-hydroxypiperidine	129.1154	$C_7H_{15}NO$	130.1232	130.1231	-0.77
7	2-Methylindole	131.0735	C_9H_9N	132.0813	132.0811	-1.51
8	Cinnamaldehyde	132.0575	C_9H_8O	133.0653	133.065	-2.25
9	3-ethyl-4-methyl-pyrrole-2,5-dione	139.0633	$C_7H_9NO_2$	140.0712	140.0709	-2.14
10	2-Phenyl-2-butenal	146.0732	$C_{10}H_{10}O$	147.0810	147.0807	-2.04
11	myosmine	146.0844	$C_9H_{10}N_2$	147.0922	147.0920	-1.36
12	5-Hydroxy-2-methylindole	147.0684	C_9H_9NO	148.0762	148.0763	0.68
13	Nornicotine	148.1000	$C_9H_{12}N_2$	149.1079	149.1075	-2.68
14	Vanillin	152.0473	$C_8H_8O_3$	153.0552	153.0551	-0.65
15	Nornicotyrine	158.0844	$C_{10}H_{10}N_2$	159.0922	159.0919	-1.89
16	Anatabiene	160.2200	$C_{10}H_{12}N_2$	161.1079	161.1077	-1.24
17	5，6-Dimethyl-1H-benzo[d]imidazol-2(3H)-one	162.0793	$C_9H_{10}N_2O$	163.0871	163.0875	1.84
18	Nicotine	162.2360	$C_{10}H_{14}N_2$	163.1235	163.1232	-1.84
19	3,4-Dimethoxybenzaldehyde	166.0630	$C_9H_{10}O_3$	167.0708	167.0707	-0.60
20	Cotinine	176.0950	$C_{10}H_{12}N_2O$	177.1028	177.1026	-1.13
21	5-(1-piperidyl)furan-2-carbaldehyde	179.0946	$C_{10}H_{13}$NO2	180.1025	180.1024	-0.56
22	Syringlyethene	180.0786	$C_{10}H_{12}O_3$	181.0865	181.0862	-1.66

Table S2. Smoke extracts components（DCM, ESI）

Compound number	Compound name	Relative molecular weight	Molecular Formula	Calculated $[M+H]^+$	Measured $[M+H]^+$	Mass error δ ($\times 10^{-6}$)
1	Benzaldehyde	106.0419	C_7H_6O	107.0497	107.0494	-2.80
2	Pyridine-3-aldehyde	107.0371	C_6H_5NO	108.0449	108.0446	-2.78
3	2,6-Lutidine	107.0735	C_7H_9N	108.0813	108.081	-2.78
4	Phenethyl alcohol	122.0732	$C_8H_{10}O$	123.0810	123.0807	-2.44

5	3-Ethyl-4-methyl-3-pyrrolin-2-one	125.0841	$C_7H_{11}NO$	126.0919	126.0916	-2.38
6	1-Ethyl-3-hydroxypiperidine	129.1154	$C_7H_{15}NO$	130.1232	130.123	-1.54
7	2-Methylindole	131.0735	C_9H_9N	132.0813	132.081	-2.27
8	Cinnamaldehyde	132.0575	C_9H_8O	133.0653	133.0651	-1.50
9	3-ethyl-4-methyl-pyrrole-2,5-dione	139.0633	$C_7H_9NO_2$	140.0712	140.071	-1.43
10	2-Phenyl-2-butenal	146.0732	$C_{10}H_{10}O$	147.0810	147.0806	-2.72
11	myosmine	146.0844	$C_9H_{10}N_2$	147.0922	147.0920	-1.36
12	5-Hydroxy-2-methylindole	147.0684	C_9H_9NO	148.0762	148.076	-1.35
13	Nornicotine	148.1000	$C_9H_{12}N_2$	149.1079	149.1075	-2.68
14	Vanillin	152.0473	$C_8H_8O_3$	153.0552	153.0551	-0.65
15	Nornicotyrine	158.0844	$C_{10}H_{10}N_2$	159.0922	159.0919	-1.89
16	Anatabiene	160.2200	$C_{10}H_{12}N_2$	161.1079	161.1077	-1.24
17	5，6-Dimethyl-1H-benzo[d]imidazol-2(3H)-one	162.0793	$C_9H_{10}N_2O$	163.0871	163.0868	-1.84
18	Nicotine	162.2360	$C_{10}H_{14}N_2$	163.1235	163.1232	-1.84
19	3,4-Dimethoxybenzaldehyde	166.0630	$C_9H_{10}O_3$	167.0708	167.0709	0.60
20	Cotinine	176.0950	$C_{10}H_{12}N_2O$	177.1028	177.1026	-1.13
21	5-(1-piperidyl)furan-2-carbaldehyde	179.0946	$C_{10}H_{13}NO2$	180.1025	180.1023	-1.11
22	Syringlyethene	180.0786	$C_{10}H_{12}O_3$	181.0865	181.0862	-1.66

Table S3. Smoke extracts components（ETOH, MPT）

Compound number	Compound name	Relative molecular weight	Molecular Formula	Calculated $[M+H]^+$	Measured $[M+H]^+$	Mass error δ ($\times10^{-6}$)
1	Benzaldehyde	106.0419	C_7H_6O	107.0497	107.0493	-3.74
2	Pyridine-3-aldehyde	107.0371	C_6H_5NO	108.0449	108.0446	-2.78
3	2,6-Lutidine	107.0735	C_7H_9N	108.0813	108.0811	-1.85
4	Phenethyl alcohol	122.0732	$C_8H_{10}O$	123.0810	123.0807	-2.44
5	3-Ethyl-4-methyl-3-pyrrolin-2-one	125.0841	$C_7H_{11}NO$	126.0919	126.0916	-2.38
6	1-Ethyl-3-hydroxypiperidine	129.1154	$C_7H_{15}NO$	130.1232	130.1231	-0.77
7	2-Methylindole	131.0735	C_9H_9N	132.0813	132.0811	-1.51
8	Cinnamaldehyde	132.0575	C_9H_8O	133.0653	133.0652	-0.75
9	3-ethyl-4-methyl-pyrrole-2,5-dione	139.0633	$C_7H_9NO_2$	140.0712	140.0711	-0.71

10	2-Phenyl-2-butenal	146.0732	$C_{10}H_{10}O$	147.0810	147.0806	-2.72
11	myosmine	146.0844	$C_9H_{10}N_2$	147.0922	147.0920	-1.36
12	5-Hydroxy-2-methylindole	147.0684	C_9H_9NO	148.0762	148.0761	-0.68
13	Nornicotine	148.1000	$C_9H_{12}N_2$	149.1079	149.1075	-2.68
14	Vanillin	152.0473	$C_8H_8O_3$	153.0552	153.0549	-1.96
15	Nornicotyrine	158.0844	$C_{10}H_{10}N_2$	159.0922	159.0920	-1.26
16	Anatabiene	160.2200	$C_{10}H_{12}N_2$	161.1079	161.1078	-0.62
17	5，6-Dimethyl-1H-benzo[d]imidazol-2(3H)-one	162.0793	$C_9H_{10}N_2O$	163.0871	163.0873	1.23
18	Nicotine	162.2360	$C_{10}H_{14}N_2$	163.1235	163.1233	-1.23
19	3,4-Dimethoxybenzaldehyde	166.0630	$C_9H_{10}O_3$	167.0708	167.0709	0.60
20	Cotinine	176.0950	$C_{10}H_{12}N_2O$	177.1028	177.1025	-1.69
21	5-(1-piperidyl)furan-2-carbaldehyde	179.0946	$C_{10}H_{13}$NO2	180.1025	180.1021	-2.22
22	Syringlyethene	180.0786	$C_{10}H_{12}O_3$	181.0865	181.0862	-1.66

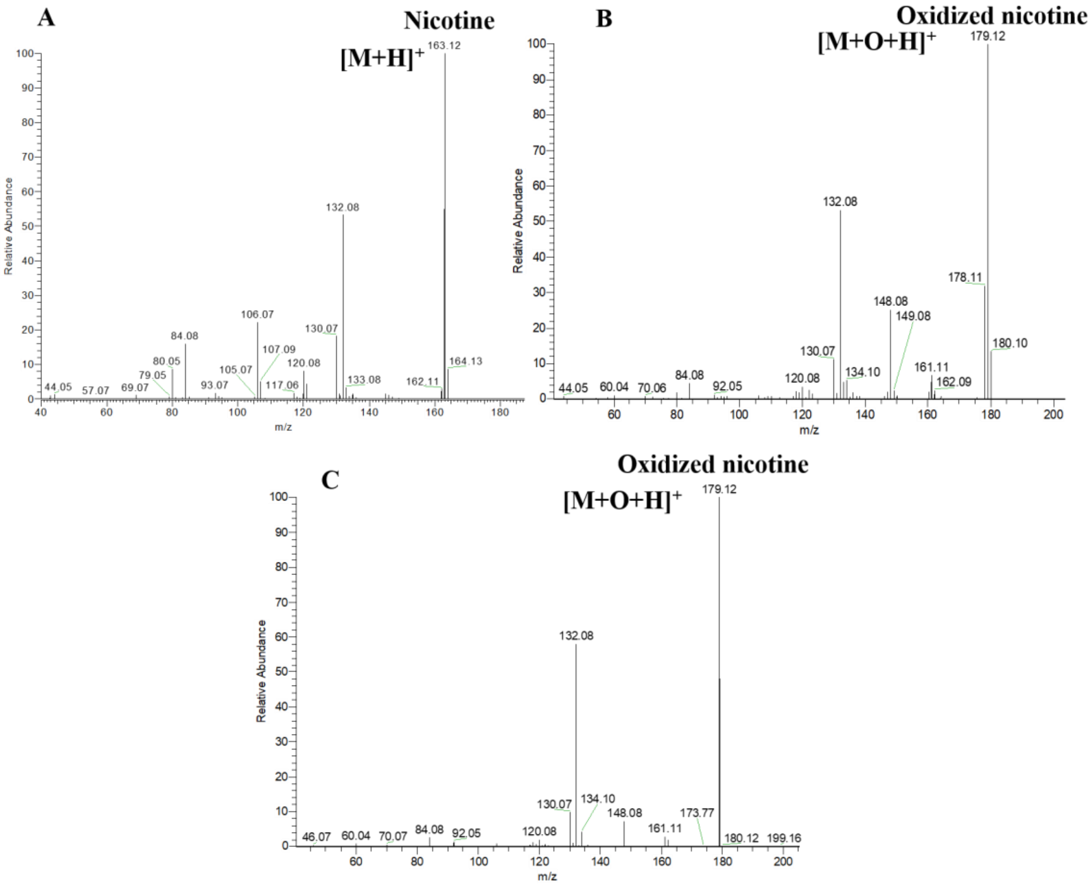

Figure S1. Spectrum of (A) MS^2 of nicotine. (B) MS^2 of nicotine oxidized product in MPT-MS. (C) spectrum of MS^2 of nicotine oxidized product in ESI-MS.

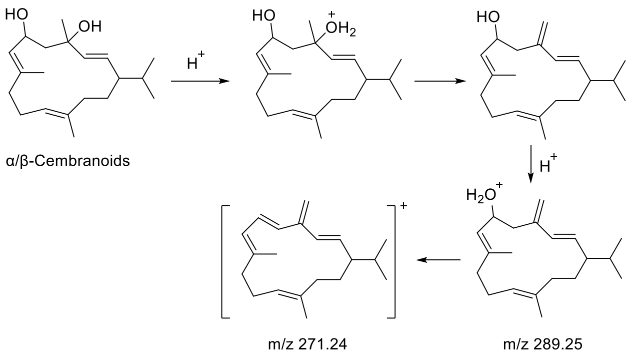

Figure S2. Molecular ion reactions of α/β-cembranoids

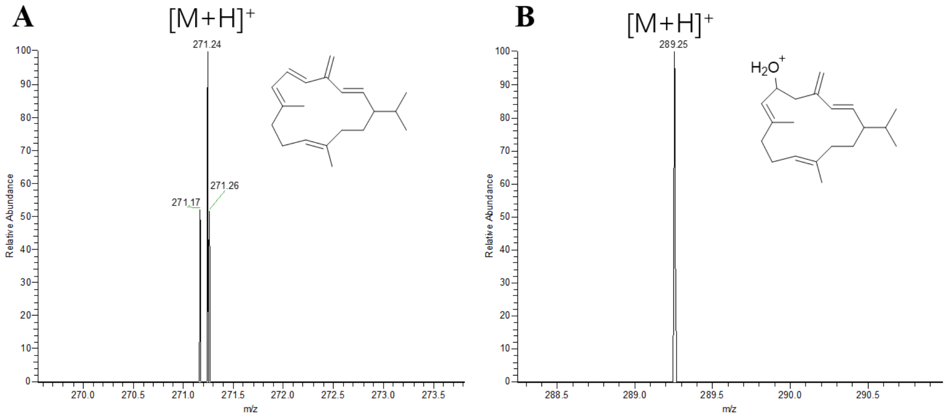

Figure S3. Spectra of α/β-cembranoids characteristic fragments (A) m/z 271.24 fragment (B). m/z 289.25 fragment.

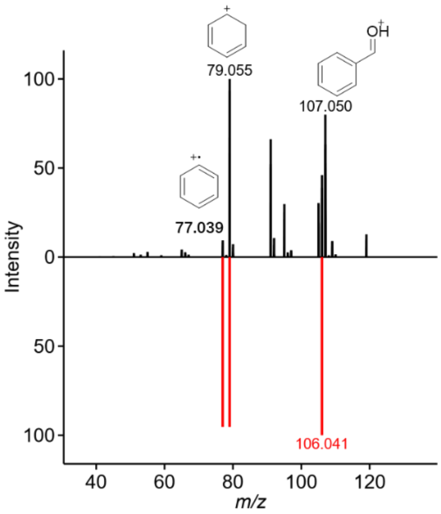

Figure S4. MS^2 spectra of Benzaldehyde(MS-Finder score: 6.96/10).

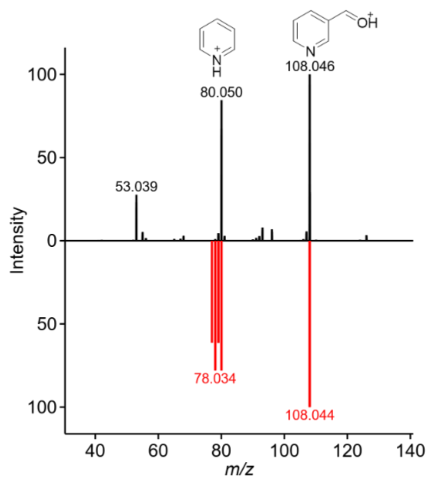

Figure S5. MS[2] spectra of Pyridine-3-aldehyde (MS-Finder score: 6.16/10).

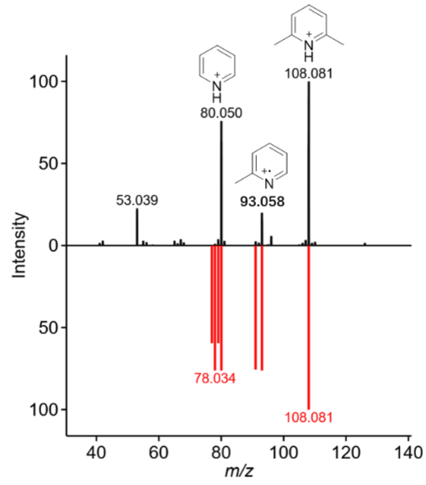

Figure S6. MS[2] spectra of 2,6-Lutidine (MS-Finder score: 6.42/10).

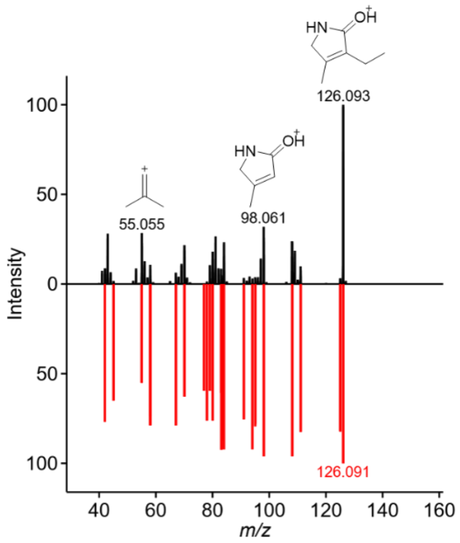

Figure S7. MS[2] spectra of 3-Ethyl-4-methyl-3-pyrrolin-2-one (MS-Finder score: 6.67/10).

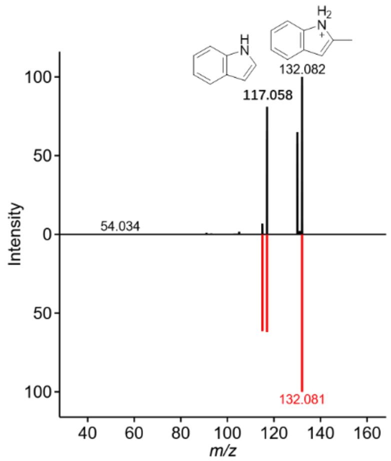

Figure S8. MS[2] spectra of 2-Methylindole (MS-Finder score: 6.68/10).

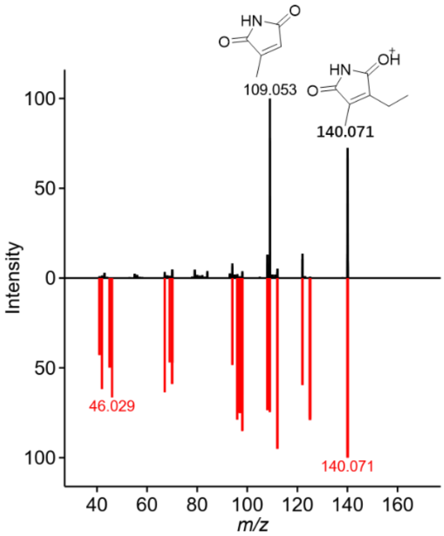

Figure S9. MS[2] spectra of 3-ethyl-4-methyl-pyrrole-2,5-dione (MS-Finder score: 6.56/10).

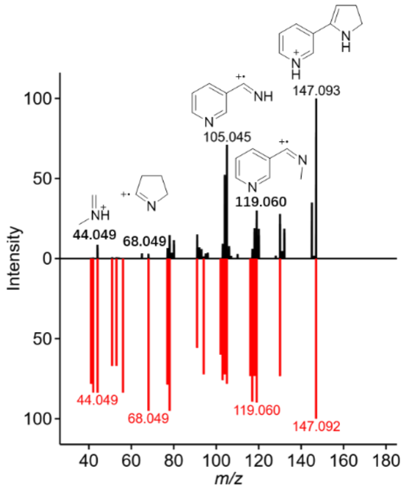

Figure S10. MS[2] spectra of Myosmine (MS-Finder score: 6.54/10).

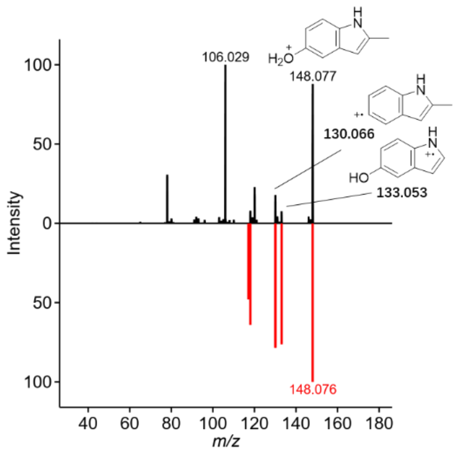

Figure S11. MS^2 spectra of 5-Hydroxy-2-methylindole (MS-Finder score: 6.49/10).

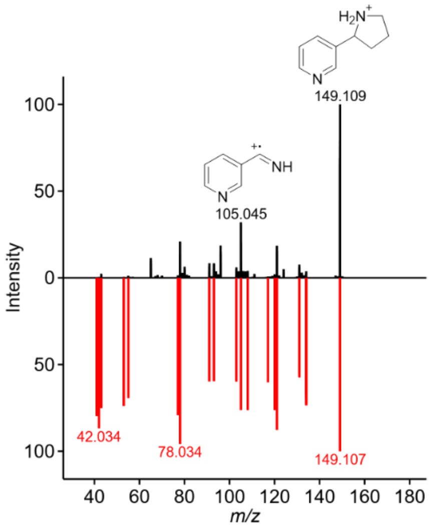

Figure S12. MS^2 spectra of Nornicotine (MS-Finder score: 7.04/10).

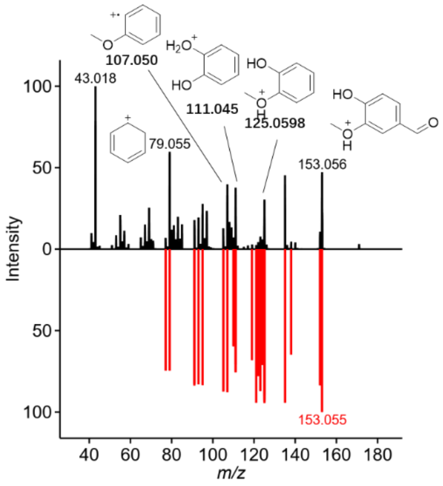

Figure S13. MS^2 spectra of Vanillin (MS-Finder score: 7.87/10).

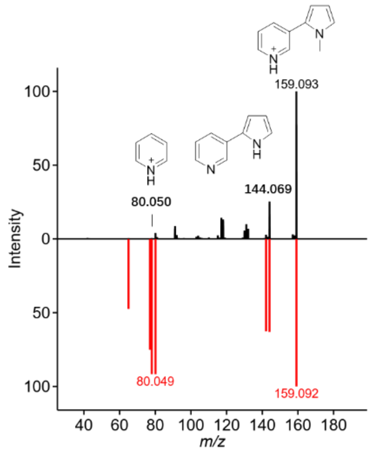

Figure S14. MS^2 spectra of Nicotyrine (MS-Finder score: 6.61/10).

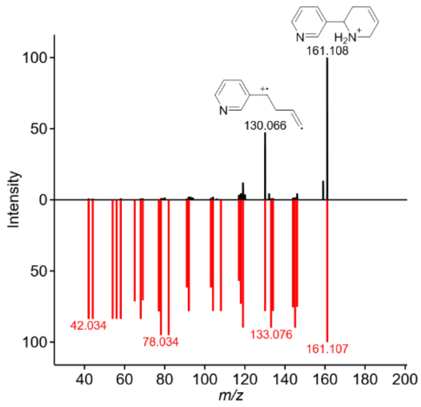

Figure S15. MS^2 spectra of Anatabine (MS-Finder score: 7.51/10).

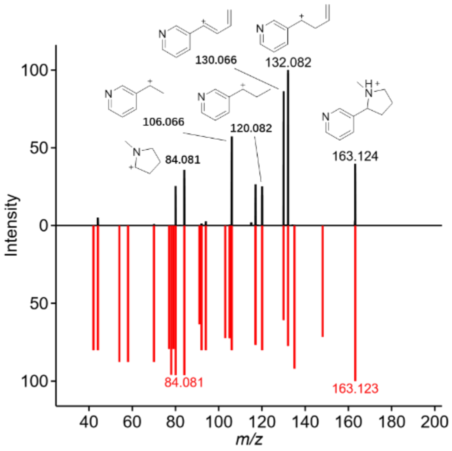

Figure S16. MS^2 spectra of Nicotine (MS-Finder score: 8.23/10).

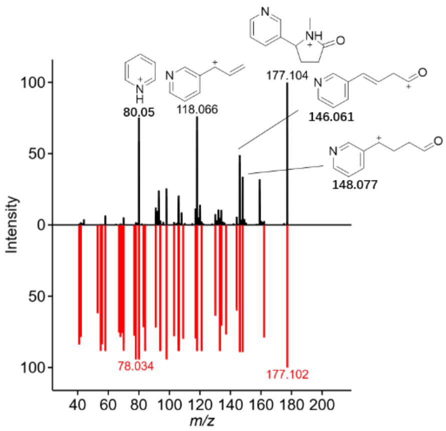

Figure S17. MS^2 spectra of Cotinine (MS-Finder score: 8.14/10).
